# Caffeine Delays Ethanol-Induced Sedation in *Drosophila*

**DOI:** 10.3390/biology12010063

**Published:** 2022-12-30

**Authors:** Sonia Tremblay, Yanqiqi Zeng, Aixin Yue, Kiana Chabot, Abigail Mynahan, Stephanie Desrochers, Sarra Bridges, S. Tariq Ahmad

**Affiliations:** 1 Department of Biology, Colby College, Waterville, ME 04901, USA; 2 Department of Psychology and Neuroscience, University of Colorado Boulder, Boulder, CO 80310, USA; 3 Department of Neurobiology, Northwestern University, Evanston, IL 60208, USA; 4 Department of Molecular Biology, Massachusetts General Hospital, Boston, MA 02114, USA; 5 New York Institute of Technology College of Osteopathic Medicine, Glen Head, NY 11545, USA; 6 Department of Psychological and Brain Sciences, Dartmouth College, Hanover, NH 03755, USA

**Keywords:** caffeine, adenosine receptor, ethanol, sedation, *Drosophila*

## Abstract

**Simple Summary:**

Caffeine and ethanol are among the most commonly consumed legal psychoactive substances worldwide. They are primarily administered in beverages for arguably opposite physiological effects—caffeine is consumed as a stimulant, and ethanol is consumed as a depressant/sedative. Both caffeine and ethanol influence many biochemical pathways including adenosine receptor-mediated signaling. Caffeine is an antagonist of adenosine receptors and ethanol elevates adenosine levels, which promotes sleep. It is important to study the interaction between caffeine and alcohol because both are easily accessible and are frequently consumed together, especially by young adults. Studies on humans have found that simultaneous intake of caffeinated drinks and alcohol increases the likelihood of alcohol consumption. In this study, using fruit flies *Drosophila melanogaster* as a model, we show that flies raised on a caffeine-supplemented diet for as little as one day or with mutation in adenosine receptor take longer to sedate when exposed to ethanol vapors. Further research in flies, which is an excellent model for behavioral studies, on the interaction between caffeine and ethanol will improve our understanding of the biochemical effect of pharmacological and psychoactive substances on behaviors associated with alcohol use disorders.

**Abstract:**

Caffeine and ethanol are among the most widely available and commonly consumed psychoactive substances. Both interact with adenosine receptor-mediated signaling which regulates numerous neurological processes including sleep and waking behaviors. In mammals, caffeine is an adenosine receptor antagonist and thus acts as a stimulant. Conversely, ethanol is a sedative because it promotes GABAergic neurotransmission, inhibits glutamatergic neurotransmission, and increases the amount of adenosine in the brain. Despite seemingly overlapping interactions, not much is known about the effect of caffeine on ethanol-induced sedation in *Drosophila*. In this study, using *Drosophila melanogaster* as a model, we show that caffeine supplementation in food delays the onset of ethanol-induced sedation in males and females of different strains. The resistance to sedation reverses upon caffeine withdrawal. Heterozygous adenosine receptor mutant flies are resistant to sedation. These findings suggest that caffeine and adenosine receptors modulate the sedative effects of ethanol in *Drosophila*.

## 1. Introduction

Caffeine is a widely consumed natural stimulant found in coffee, tea, and chocolate [1,2]. In the United States, individuals consume an estimated daily average of 165–170 mg of caffeine [3,4] and an estimated 10 billion kg of coffee was consumed worldwide in 2020–21 [5]. Caffeine (1,3,7-trimethylxanthine) is an alkaloid with structural similarities to purine nucleic acids. Neurally, caffeine has multiple proposed mechanisms of action, including intracellular calcium mobilization, cAMP phosphodiesterase inhibition, and adenosine receptor antagonism [2,6]. Caffeine is an antagonist of all four subtypes of mammalian adenosine receptors (A_1_, A_2A_, A_2B_, and A_3_) but the antagonism of the A_1_ and A_2A_ receptors is most well characterized [7,8]. *Drosophila* has a single adenosine receptor subtype which is most similar to the mammalian A_2A_ subtype of adenosine receptor. *Drosophila* adenosine receptor is expressed in all developmental stages with the highest expression observed in the adult head [9]. Adenosine receptors are G-protein coupled receptors (GPCRs) and primarily function through cAMP and PKA signaling pathways for downstream functionality [9].

Adenosine is a nucleoside produced during the breakdown of ATP [10], which acts as a neuromodulator performing multiple functions related to reducing neuronal excitability in the central nervous system [11]. Adenosine signaling promotes sleep by inhibiting wakefulness-promoting neurons [12,13]. Neuronal production of adenosine is also higher during waking hours than during sleep, allowing the promotion of wakefulness as morning approaches [12,14].

Ethanol is another widely consumed psychoactive substance that acts as a depressant and promotes sleep [15]. Ethanol has multiple mechanisms of action, contributing to these behavioral effects, which include promoting GABA signaling and inhibiting glutamatergic signaling [16]. Additionally, ethanol interacts with adenosine neurotransmission to exert somnogenic effects, potentially by increasing the amount of adenosine available to target A_1_ receptors [16,17].

*Drosophila melanogaster* serves as an ideal animal model for studying the effects of psychoactive compounds on behavior [18,19]. Upon exposure to ethanol, *Drosophila* exhibits analogous responses to humans including biphasic hyperactivity and sedation, tolerance, and preference for alcohol using similar biochemical pathways [20,21]. Furthermore, *Drosophila* and vertebrates share many of the metabolic pathways implicated in the behavioral response to ethanol [21]. To date, no studies have characterized the effect of caffeine and adenosine receptors on ethanol-induced sedation in flies. Here, we demonstrate that caffeine and heterozygous adenosine receptor mutation delay the onset of ethanol-induced sedation in *Drosophila*.

## 2. Materials and Methods

### 2.1. Drosophila Stocks and Maintenance

*Drosophila* stocks *w^1118^* (provided by Joshua Kavaler, Colby College, Waterville, ME, USA), *OR-R* (Bloomington stock #5), and *AdoR^MB04401^* (Bloomington stock #24699) were obtained from the Bloomington *Drosophila* Stock Center (Bloomington, IN, USA). *w^1118^* flies were used as control flies because the adenosine receptor mutant allele was generated in the *w^1118^* background. The fly stocks were maintained on standard Nutri-Fly^TM^ Bloomington Formulation (Genesee Scientific, San Diego, CA, USA). In all trials, light conditions, (12 h light/dark cycle), environmental temperature (25 °C), food type, and time of day of sedation were kept constant unless otherwise stated to minimize the chance of conflicting variables. Sedation assays were carried out between ZT6 and ZT10.

### 2.2. Food Preparation

Each food vial contained 1.5 g of Nutri-Fly^TM^ Instant Formulation (Genesee Scientific, San Diego, CA, USA) instant food (Genesee Scientific) and 7.5 mL distilled water, which swells to 10 mL volume. Caffeine (Sigma, St. Louis, MO, USA) was dissolved in distilled water and mixed with instant food to make supplemented food (caffeine—0.25 mg/mL, 0.5 mg/mL, and 1.0 mg/mL).

### 2.3. Caffeine Supplementation

Flies (1–3 days old) were raised on caffeine-supplemented food for the specified experimental timeline followed by ethanol-induced sedation assay.

For dose-dependency experiments (both caffeine and ethanol) and withdrawal experiments, flies were raised on caffeine-supplemented food for three days.

For variable exposure length studies, time on caffeine-supplemented food ranged from one to five days.

For sustainability of effect (withdrawal) experiments, flies were placed in vials containing non-caffeinated food for a designated period of time ranging from one to three days. Flies were age-matched such that they were all the same age at the time of the sedation assays.

### 2.4. Caffeine-Induced Mortality

The surviving flies were counted after the full period of supplementation with caffeine (three or five days) to calculate mortality rates.

### 2.5. Ethanol-Induced Sedation Assay

A modified ethanol sedation assay from Maples and Rothenfluh was followed as previously reported [22,23]. Male or female flies (8–10) were sedated using CO_2_ and sorted into empty vials. Flies were allowed to recover from the effects of CO_2_ anesthesia for 0.5–8 h before sedation assay. To determine time of sedation, the cotton plug of each vial was replaced with a new plug soaked with 500 μL of the appropriate dose (25%, 50%, 75%, and 100% *v*/*v*) of 200-proof ethanol (Fisher Scientific). After one minute, each vial was tapped once on the table to relocate the flies to the bottom of the vial. After ten seconds, the number of flies exhibiting loss-of-righting reflex were recorded. This procedure was repeated after every minute of ethanol exposure for each vial until at least half of the flies in the vial were sedated. Loss of righting reflex includes flies on their backs, stationary flies, stationary flies with rapid wing movement, and spinning in one location. The number of minutes it took for half the flies in the vial to sedate was recorded for each vial as the median sedation time (ST50). Linear interpolation was used to determine ST50 when there was an odd number of flies in the trial or when 50% sedation was reached in between intervals.

### 2.6. Statistical Analysis

Multiple two-tailed independent samples *t*-tests and one-way ANOVAs with Tukey HSD post hoc tests were conducted using RStudio.

## 3. Results

### 3.1. Caffeine Delays Onset of Ethanol-Induced Sedation in Wild Type Flies

To determine the effect of caffeine on ethanol-induced sedation, we performed sedation assays (with 100% ethanol), which rely on loss-of-righting reflex, on 1–3 day-old male and female *w^1118^* flies, a widely used control strain, after exposing them to caffeine-supplemented food for three days.

**Figure 1:**
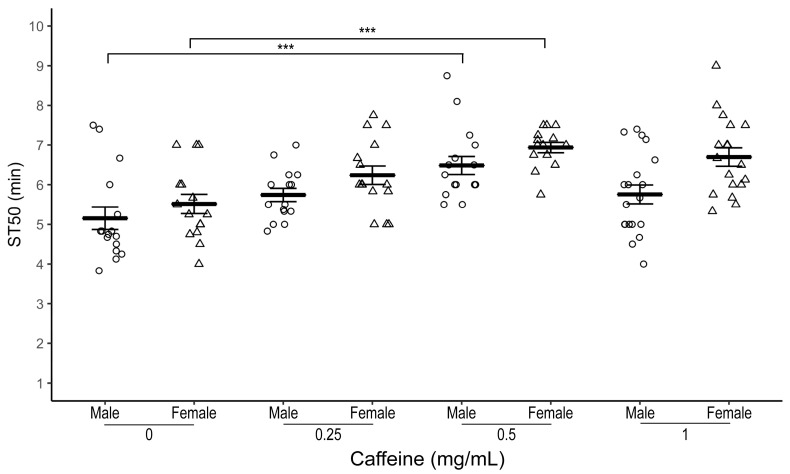
Effect of caffeine supplementation on ethanol-induced sedation in *w^1118^* flies. Comparison of ST50 when exposed to 100% ethanol of 1–3 day old male (circles) and female (triangles) *w^1118^* flies raised for three days at different dosages (0.25, 0.5, and 1 mg/mL) of caffeine-supplemented food. Both male and female *w^1118^* flies raised on 0.5 mg/mL caffeine-supplemented food have a significantly higher ST50 than control flies. Horizontal bars represent the mean ST50 ± standard error of 15–19 biological replicates. For each sedation assay 8–10 male flies were used. *** *p* = 0.001; ANOVA, post hoc Tukey HSD.

Male flies raised on 0.5 mg/mL caffeine-supplemented food had a significantly higher median sedation time (ST50) compared to control flies with no caffeine supplementation (Figure 1; average ST50_caff_ = 6.49 ± 0.23 min, ST50_cont_ = 5.15 ± 0.28 min, *n* = 16 biological replicates, one-way ANOVA-Tukey, *p* = 0.001). Flies exposed to other doses of caffeine (0.25 mg/mL, *n* = 15 biological replicates or 1.0 mg/mL caffeine, *n* = 19 biological replicates) did not have a statistically significant different ST50 than control flies. However, we observed a trend for higher ST50 in lower doses of caffeine compared to control flies (Figure 1). The delay in onset of sedation at 0.5 mg/mL caffeine dose was more pronounced when sedation assays were performed with 50% and 75% ethanol (Table 1 and Figure S1).

Similarly, female *w^1118^* flies showed the most robust increase in ST50 at 0.5 mg/mL caffeine concentration (Figure 1; average ST50_caff_ = 6.94 ± 0.13 min, ST50_cont_ = 5.51 ± 0.24 min, *n* = 14–15 biological replicates, one-way ANOVA-Tukey HSD, *p* = 0.0001).

The mortality was not statistically significant for any caffeine dose in either males or females. However, we observed a trend of increased mortality in both males and females in a dose- and duration of exposure-dependent manner, with the highest mortality occurring in flies exposed to 1.0 mg/mL caffeine for five days (Supplementary Table S1).

**Figure 2:**
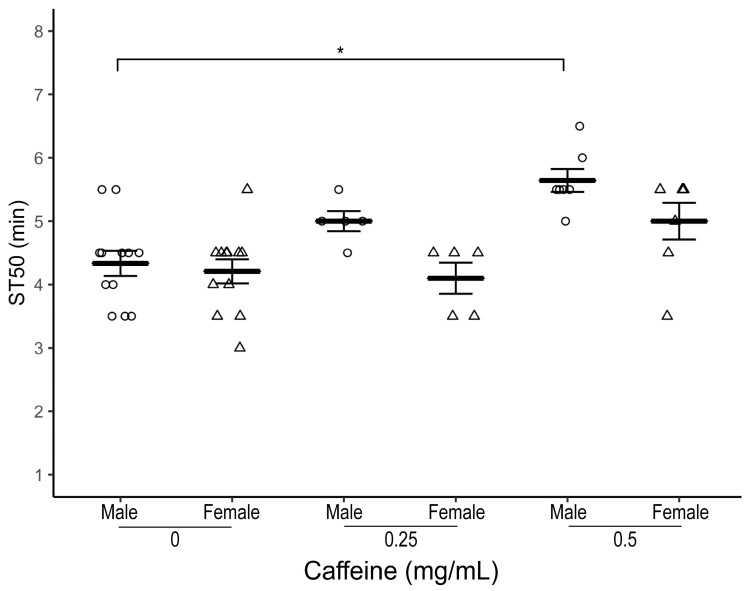
Effect of caffeine supplementation on ethanol-induced sedation in wildtype (*OR-R*) flies. Comparison of ST50 when exposed to 100% ethanol of 1–3 day old male (circles) and female (triangles) *OR-R* flies raised for three days at different dosages (0.25 and 0.5 mg/mL) of caffeine-supplemented food. Both male and female flies raised on 0.5 mg/mL caffeine-supplemented food for three days have the most elevated ST50 than control flies (*p* = 0.04 (males), *p* = 0.06 (females)). Horizontal bars represent the mean ST50 ± standard error of 5–12 biological replicates. For each ST50 8–10 male and female flies were used. * *p* < 0.05; ANOVA, post hoc Tukey HSD.

We then tested the effect of caffeine on ethanol-induced sedation in *OR-R*, a commonly used wildtype strain of *Drosophila*. Both male and female *OR-R* flies raised on caffeine-supplemented food (0.25 and 0.5 mg/mL) for three days had a higher ST50 at all caffeine doses with the most robustly elevated ST50 observed at 0.5 mg/mL dose compared to control flies (Figure 2; Males—average ST50_caff_ = 5.64 ± 0.18 min, ST50_cont_ = 4.5 ± 0.20 min, *n* = 7–12 biological replicates, one-way ANOVA-Tukey HSD, *p* = 0.04 and Females—average ST50_caff_ = 5.0 ± 0.29 min, ST50_cont_ = 4.21 ± 0.19 min, *n* = 7–12 biological replicates, one-way ANOVA-Tukey HSD, *p* = 0.06).

### 3.2. Exposure to Caffeine for One Day Is Sufficient to Increase Ethanol-Induced Sedation Time

**Figure 3:**
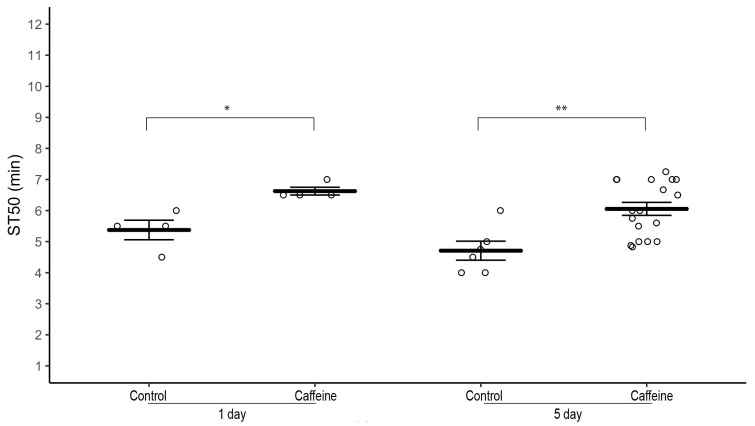
Effect of time course of caffeine supplementation on ethanol-induced sedation in *w^1118^* flies. Male *w^1118^* flies (1–3 day old) raised on 0.5 mg/mL caffeine-supplemented food for one day and five days have a significantly higher ST50 when exposed to 100% ethanol than control flies. Horizontal bars represent the mean ST50 ± standard error of 4–18 biological replicates. For each sedation assay 8–10 male flies were used (circles). * *p* < 0.05, ** *p* < 0.01; two-tailed independent samples *t*-test.

To test if the effects of caffeine on sedation time depend on the duration of its exposure, we did a time-course of caffeine exposure by raising 1–3 day-old *w^1118^* flies on 0.5 mg/mL caffeine-supplemented food for one and five days. Male and female flies exposed to caffeine for both one day and five days had a significantly higher ST50 than corresponding control males and females, respectively (Figure 3; 1-day: Males—average ST50_caff_ = 6.63 *±* 0.12 min, ST50_cont_ = 5.38 min *±* 0.31, *n* = 4 biological replicates, two-tailed independent samples *t*-test, *p* = 0.022 and Females (Figure S2)—average ST50_caff_ = 6.13 *±* 0.24 min, ST50_cont_ = 5.00 *±* 0.35 min, *n* = 4 biological replicates, two-tailed independent samples *t*-test, *p* = 0.044. 5-day: Males—average ST50_caff_ = 6.05 *±* 0.21 min, ST50_cont_ = 4.71 *±* 0.30 min, *n* = 6–18 biological replicates, two-tailed independent samples *t*-test, *p* = 0.002 and Females—average ST50_caff_ = 6.50 *±* 0.2 min, ST50_cont_ = 4.88 *±* 0.28 min, *n* = 6–20 biological replicates, two-tailed independent samples *t*-test, *p* < 0.001).

Unless specified otherwise, for all subsequent experiments data from 1–3 day-old male *w^1118^* flies raised on 0.5 mg/mL caffeine-supplemented food for three days is presented.

### 3.3. The Effect of Caffeine on Ethanol-Induced Sedation Reverses after Caffeine Withdrawal

To determine the lasting effect of caffeine-mediated resistance to sedation upon cessation of exposure to caffeine, 1–3 day-old male *w^1118^* flies were raised on 0.5 mg/mL caffeine-supplemented food for three days and subsequently transferred to non-caffeinated food for one day or three days.

**Figure 4:**
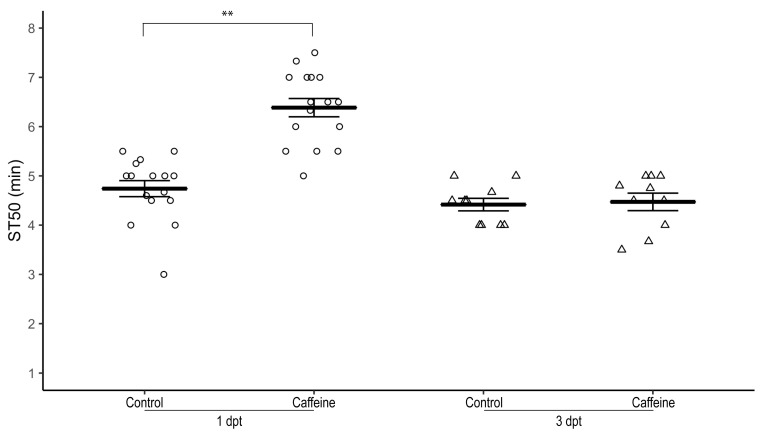
Sustainability of caffeine-mediated effect on ethanol-induced sedation after caffeine withdrawal. Male *w^1118^* flies raised on 0.5 mg/mL caffeine-supplemented food for three days have a significantly higher ST50 when exposed to 100% ethanol than control flies (*p* < 0.001) one day post caffeine withdrawal (circles) but the ST50 three days post caffeine withdrawal (triangles) is not significantly different (*p* = 0.804). Horizontal bars represent the mean ST50 ± standard error of 10–16 biological replicates. For each sedation assay 8–10 male flies were used. dpt—days post-transfer to control food; ** two-tailed independent samples *t*-test.

At one day post-transfer to regular food, both male (Figure 4) and female (not shown) flies had a significantly higher ST50 compared to control male and female flies, respectively (average ST50_caff_ = 6.39 ± 0.19 min, ST50_cont_ = 4.74 ± 0.16 min, *n* = 16 biological replicates, two-tailed independent samples *t*-test, *p* < 0.01).

At three days post-transfer to regular food, ST50 of male (Figure 4) and female (not shown) flies was not significantly different from control flies (average ST50_caff_ = 4.47 ± 0.13 min, ST50_cont_ = 4.42 ± 0.18 min, *n* = 10 biological replicates, two-tailed independent samples *t*-test, *p* = 0.804).

Taken together, these data show that caffeine delays onset of sedation in male and female wild type flies that can initiate after exposure to caffeine for one day and lasts up to three days after caffeine withdrawal.

### 3.4. Adenosine Receptor Mutation Delays Onset of Ethanol-Induced Sedation

**Figure 5:**
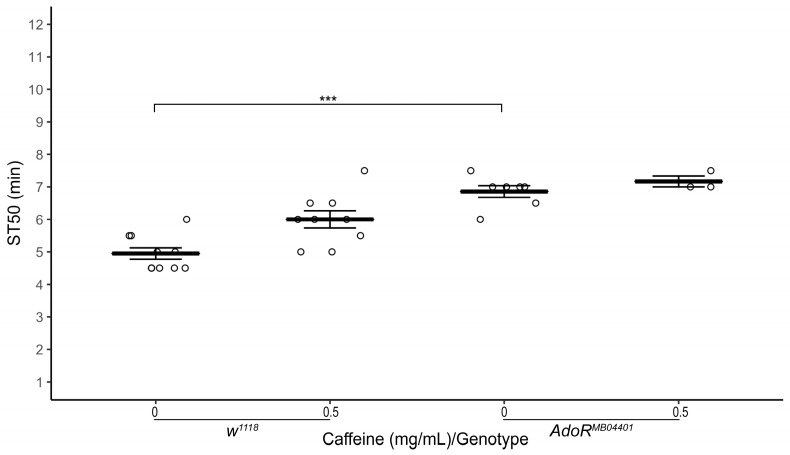
Effect of adenosine receptor mutation on ethanol-induced sedation. Comparison of ST50 when exposed to 100% ethanol of 1–3 day old male adenosine receptor mutant flies (*AdoR^MB04401^/TMC, Sb*) and *w^1118^* flies raised for three days on (0 mg/mL and 0.5 mg/mL) caffeine-supplemented food. Heterozygous *AdoR* mutant flies (*AdoR^MB04401^*/*TM6C, Sb*) raised on control food and 0.5 mg/mL caffeine-supplemented food have a significantly higher ST50 than *w^1118^* flies raised on control (*p* < 0.001) and 0.5 mg/mL caffeine-supplemented food (*p* = 0.004), respectively. Horizontal bars represent the mean ST50 ± standard error of 3–9 biological replicates. For each sedation assay 8–10 male flies were used (circles). *** *p* < 0.001; two-tailed independent samples *t*-test.

In mammals, caffeine is an adenosine receptor antagonist [6,7,24]. Therefore, we wondered if adenosine receptor mutant flies are resistant to sedation. Indeed, heterozygous adenosine receptor mutant flies (*AdoR^MB04401^*/*TM6C*, *Sb*) raised on normal food showed a significantly higher ST50 compared to *w^1118^* flies (Figure 5; average ST50*_AdoR+/−_* = 6.86 ± 0.18 min, ST50*_w1118_* = 4.95 ± 0.17 min, *n* = 7–10 biological replicates, two-tailed independent samples *t*-test, *p* < 0.001).

## 4. Discussion

*Drosophila* is an excellent model to study the effects of caffeine, adenosine receptor signaling, and alcohol on a variety of behaviors such as circadian rhythm, locomotion, and cognition [9,13,22,25]. This study, to the best of our knowledge, is the first to examine interplay among caffeine, *Drosophila* adenosine receptor, and ethanol-induced sedation in *Drosophila*. Our results demonstrate that exposure to caffeine prolongs the onset of sedation in both male and female *w^1118^* and *OR-R* flies, commonly used control and wild type *Drosophila* strains, respectively. Sex differences in ethanol-induced sedation time are observed in both vertebrates and invertebrates including *Drosophila*. Female flies are reported to have a shorter sedation time than males [26]. However, in *w^1118^* sexual dimorphism of sedation does not resolve when exposed to 100% ethanol [27]. We observed that *w^1118^* females showed a trend (not statistically significant) for a higher ST50 than males in control at all concentrations of caffeine tested.

The effect of caffeine on sedation time when sedated with 100% ethanol was most robust and statistically significant at 0.5 mg/mL dose and did not hold at 1 mg/mL dose. The effect of caffeine on sedation time was more prominent with 50% and 75% ethanol possibly because the effect of caffeine manifests more effectively when the rate of onset of sedation is less drastic at lower doses of ethanol. Other studies in flies have examined the effects of caffeine, at a similar dose range, on adenosine receptor expression, fecundity, egg laying, and life span [28,29]. It is possible that at higher doses, flies are generally weaker or less viable, as caffeine appears to begin to confer mortality in flies at 1 mg/mL concentration. We observed significant widespread mortality at 3 mg/mL and 5 mg/mL dose after one-week exposure (data not shown). At higher caffeine doses, a weakened state is possible either because flies are avoiding consuming highly caffeinated food due to its bitter taste [30,31] and/or due to the direct physiological effects of caffeine. There is support for a direct effect of caffeine on physiological functions leading to mortality in flies as shown by the correlation between caffeine-induced mortality and reduced transcript levels of neuromodulators and adenosine receptors [28,32]. The effect of caffeine on sedation is reversible and wears off within three days of caffeine withdrawal, likely due to its metabolic clearance. Caffeine is metabolized by the Cytochrome P-450 enzymes in both *Drosophila* and humans [33,34]. Caffeine metabolites (theophylline, theobromine, paraxanthine) are also detected within 3 h of caffeine ingestion in flies [33]. The half-life of caffeine in humans is 3–7 h [34].

Caffeine primarily acts as a stimulant in mammals because of its antagonism of adenosine receptor signaling, which promotes sleep [6,7,24]. In this study, we addressed the relationship between adenosine receptor gene dosage and sedation in flies. We found that heterozygous adenosine receptor mutant flies are resistant to sedation. We were unable to conduct sedation assays on homozygous mutant flies for the *AdoR^MB04401^* allele because they generally do not survive to adulthood. This finding is in accordance with a previous study in which adenosine A_2A_ receptor knockout mice were shown to be resistant to ethanol-induced hypnotic effects [35]. Mammalian adenosine A_2A_ receptor has the highest homology to the only adenosine receptor isoform in *Drosophila* [13]. However, in *Drosophila*, caffeine may also act through additional pathways, such as the dopamine receptor-mediated signaling. The fly dopamine/ecdysteroid receptor (DopEcR) mediates ethanol-induced sedation [36]. The *Drosophila* dopamine receptor (dDA1) [37] and dopamine signaling [38] have been independently shown to modulate the wake-promoting effect of caffeine, suggesting a potential adenosine receptor-independent mechanism of action for caffeine in flies. Additionally, the effects of caffeine on sleep does not depend on adenosine activity [13]. Further, caffeine was unable to inhibit adenosine receptor-mediated signaling in *Drosophila* neuroblast cell line in vitro [39]. Therefore, it appears that caffeine and adenosine receptor either through coordination and/or independently play a role in ethanol-induced sedation.

Our data suggests that pharmacological (caffeine exposure) and genetic (adenosine receptor mutation) disruption of adenosine receptor function delays ethanol sedation. Therefore, it can be hypothesized that agonists of the adenosine receptor, especially the endogenous ligand, adenosine, will promote sedation. Ethanol elevates extracellular adenosine levels which in turn activate the adenosine receptors [40]. In mammalian systems, the ethanol-mediated elevation of adenosine modulates ethanol-induced behaviors primarily through adenosine A_1_ and A_2_ receptors [11,35,40]. In future studies, it will be interesting to determine effects of adenosine receptor agonists on sedation and the cellular and molecular characteristics of signaling pathways downstream of adenosine receptors that mediate ethanol-induced sedation in flies.

## 5. Conclusions

Broadly, this study further supports the use of *Drosophila* as a model to study complex human behavior and to examine the colloquial notion of the negative implications of mixing caffeine with alcohol. Human correlational studies have found that individuals concurrently consuming caffeinated energy drinks and alcohol are more likely to consume more alcohol and have more severe negative consequences due to alcohol consumption [41]. When both substances are consumed in conjunction, caffeine reduces perceived inebriation, which leads to further consumption, thereby promoting binge drinking and risky behavior such as driving under the influence of alcohol [42]. Further studies on the interaction between caffeine and ethanol will improve our understanding of the biochemical and behavioral consequences of their consumption and aid in creating awareness of this public health crisis, especially for adolescents and young adults [43,44].

## Figures and Tables

**Table 1:** Effect of caffeine on sedation at different ethanol concentrations.

	Median Sedation Time ST50 (min)		Change in ST50 (%) Caffeine vs. Control
Ethanol (%)/Caffeine (mg/mL)	0	0.5	
50	12.12 ± 1.5 ^a,b^	20.03 ± 1.7 ^i,ii^	65.26 **
75	6.97 ± 0.5 ^a^	11.37 ± 0.9 ^i,iii^	63.13 **
100	4.66 ± 0.3 ^b^	6.23 ± 0.3 ^ii,iii^	33.70 **

*p*-values: ^a^ = 0.002; ^b,i,ii^ < 0.001; ^iii^ = 0.008; ANOVA, post hoc Tukey HSD and ** *p* < 0.01 *t*-test; *n* = 8–10.

## Data Availability

Not applicable.

## Supplementary Materials

The following supporting information can be downloaded at: https://www.mdpi.com/article/10.3390/biology12010063/s1. Figure S1. Effect on caffeine on sedation at different ethanol concentrations. Figure S2. Effect of time course of caffeine supplementation on ethanol-induced sedation in *w^1118^* flies. Table S1. Effect of caffeine on mortality in flies.
