# Supplementary material for: Caffeine Delays Ethanol-Induced Sedation in Drosophila
Source: Biology (Basel). 2022 Dec 30;12(1):63. doi: 10.3390/biology12010063 (PMC9855986; doi:10.3390/biology12010063)
Supplement: Supplementary file 1 [file biology-12-00063-s001.zip › biology-2092513-supplementary.pdf]

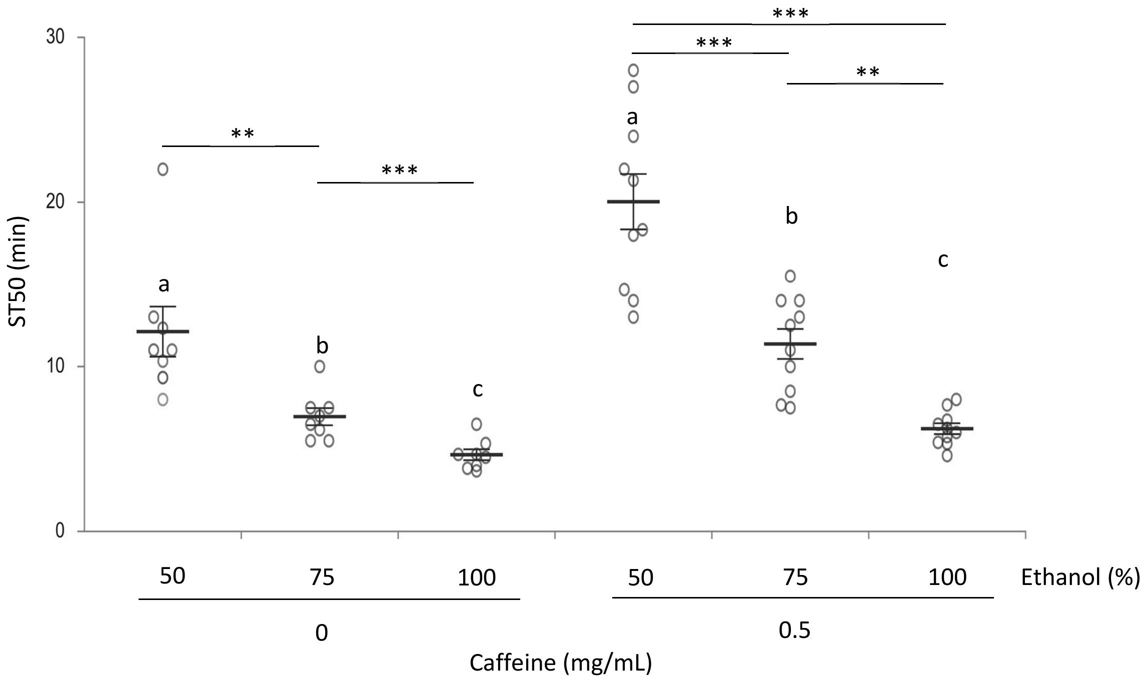

Figure S1. Effect on caffeine on sedation at different ethanol concentrations. To assess the effect of ethanol concentration on sedation, 1-3 day-old male w^{1118} flies were given food supplemented with either 0 or 0.5 mg/mL caffeine for three days. Sedation assays were then performed with 50%, 75%, and 100% ethanol on 8-10 flies each (circles). Male w^{1118} flies given 0 mg/mL caffeine (controls) sedated significantly slower with 50% ethanol than with both 75% and 100% ethanol. Although flies sedated using 75% ethanol sedated slower than those under 100% ethanol, this difference was not statistically significant, $p = 0.217$ ($ST50_{50\%} = 12.12 \pm 1.52$ min, $ST50_{75\%} = 6.97 \pm 0.51$ min, $ST50_{100\%} = 4.66 \pm 0.33$ min, $n = 8$ biological replicates, one-way ANOVA-Tukey HSD, $ST50_{50\%}$ vs $ST50_{75\%}$ $^{**}p = 0.002$, $ST50_{50\%}$ vs $ST50_{100\%}$ $^{***}p < 0.001$). Male w^{1118} flies given 0.5 mg/mL caffeine sedated significantly slower with 50% ethanol than with 75% and 100% ethanol. Flies sedated with 75% ethanol also sedated significantly slower than with 100% ethanol ($ST50_{50\%} = 20.03 \pm 1.68$ min, $ST50_{75\%} = 11.37 \pm 0.91$ min, $ST50_{100\%} = 6.23 \pm 0.33$ min, $n = 10$ biological replicates, one-way ANOVA-Tukey HSD, $ST50_{50\%}$ vs $ST50_{75\%}$ $^{***}p < 0.001$, $ST50_{50\%}$ vs $ST50_{100\%}$ $^{***}p < 0.001$, $ST50_{75\%}$ vs $ST50_{100\%}$ $^{**}p = 0.008$). These results reflect a 65.24% ($^{a}p = 0.003$, *t*-test) increase in ST50 between control and caffeinated flies at 50% ethanol, a 63.06% ($^{b}p = 0.001$, *t*-test) increase in ST50 between control and caffeinated flies at 75% ethanol, and a 33.62% ($^{c}p = 0.004$, *t*-test) increase in ST50 between control and caffeinated flies at 100% ethanol. This data is presented in Table 1 in the manuscript.

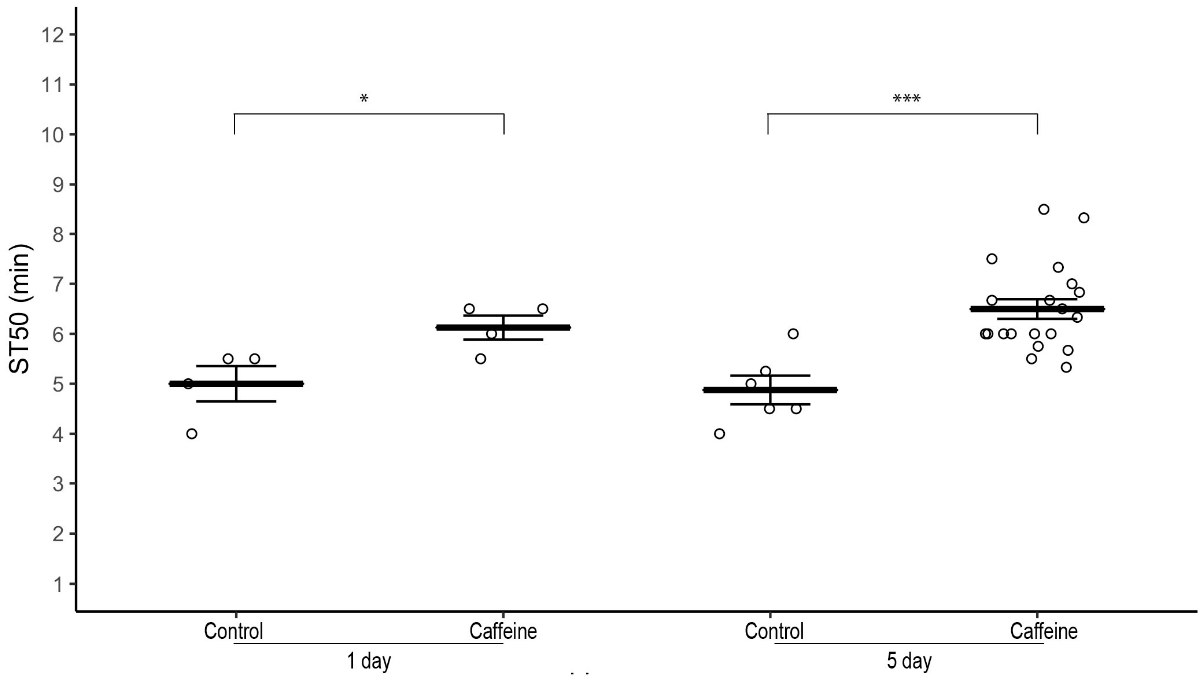

Figure S2. Effect of time course of caffeine supplementation on ethanol-induced sedation in *w*^{1118} flies. Female *w*^{1118} flies (1-3 day old) raised on 0.5 mg/mL caffeine-supplemented food for one day and five days have a significantly higher ST50 when exposed to 100% ethanol than control flies. Horizontal bars represent the mean ST50 ± standard error of 4–18 biological replicates. For each sedation assay 8-10 female flies were used (circles). *$p < 0.05$, ***$p < 0.001$; *t*-test.

Table S1. Effect of caffeine on mortality in flies

Caffeine (mg/ml)	Mortality (%) Male			
	3 day	n/batch	5 day	n/batch
0.0	0.0	92/2	2.39±1.19	282/6
0.25	0.0	89/2	2.64±0.82	225/5
0.5	0.0	91/2	5.46±1.71	204/5
1.0	0.0	98/2	18.94±10.81	237/6

Caffeine causes higher mortality at higher doses and longer exposure:

Average percent mortality per batch in males was not significantly different between any caffeine dosages in flies supplemented for 3 days or 5 days (one-way ANOVA, p_{3day} = na, p_{5day} = 0.185). However, the trend indicates that mortality in w^{1118} flies increases with caffeine dosage, with the highest rates of mortality occurring at the highest tested dosage of 1.0 mg/mL caffeine. Higher rates of mortality were observed in flies supplemented for 5 days, indicating that prolonged supplementation time increases risk of death from caffeine in w^{1118} flies, potentially due to a buildup of toxicity over time.
